# Face-to-Face and Digital Multidomain Lifestyle Interventions to Enhance Cognitive Reserve and Reduce Risk of Alzheimer’s Disease and Related Dementias: A Review of Completed and Prospective Studies

**DOI:** 10.3390/nu11092258

**Published:** 2019-09-19

**Authors:** Nicholas T. Bott, Aidan Hall, Erica N. Madero, Jordan M. Glenn, Nami Fuseya, Joshua L. Gills, Michelle Gray

**Affiliations:** 1 Clinical Excellence Research Center, Stanford University School of Medicine, Stanford, CA 94305, USA; 2 Neurotrack Technologies, Inc. Redwood City, CA 94063, USA; aidan@neurotrack.com (A.H.); erica@neurotrack.com (E.N.M.); jordan@neurotrack.com (J.M.G.); nami@neurotrack.com (N.F.); 3 Exercise Science Research Center, University of Arkansas, Fayetteville, AR 72701, USA; jgills@email.uark.edu (J.L.G.); rgray@uark.edu (M.G.)

**Keywords:** telemedicine, internet, digital, lifestyle, healthy aging, cognition, cognitive reserve, dementia, Alzheimer’s disease, health promotion, primary prevention, risk reduction

## Abstract

Background: Currently, there is no pharmaceutical intervention to treat or delay pathological cognitive decline or Alzheimer’s disease and related dementias (ADRD). Multidomain lifestyle interventions are increasingly being studied as a non-pharmacological solution to enhance cognitive reserve, maintain cognition, and reduce the risk of or delay ADRD. Review of completed and prospective face-to-face (FTF) and digital multidomain interventions provides an opportunity to compare studies and informs future interventions and study design. Methods: Electronic databases (PubMed, PsycINFO, clinicaltrials.gov and NIH RePORTER) were searched for multidomain lifestyle programs. Studies were included if the program (1) included a control group, (2) included at least 3 interventions, (3) were at least 6 months in duration, and (4) included measurement of cognitive performance as an outcome. Results: In total, 17 multidomain lifestyle programs aimed at enhancing cognitive reserve and reducing risk of ADRD were found. Thirteen programs are FTF in intervention delivery, with 3 FTF programs replicating the FINGER protocol as part of the World Wide Fingers Consortium. Four programs are delivered digitally (website, Web application, or mobile app). Program characteristics (e.g., target population, duration, frequency, outcomes, and availability) and results of completed and prospective studies are reviewed and discussed. Conclusion: This review updates and discusses completed and current multidomain lifestyle interventions aimed at enhancing cognitive reserve and reducing risk of ADRD. A growing number of international studies are investigating the efficacy and utility of these programs in both FTF and digital contexts. While a diversity of study designs and interventions exist, FTF and digital programs that build upon the foundational work of the FINGER protocol have significant potential to enhance cognitive reserve and reduce risk of ADRD.

## 1. Introduction

An estimated 50 million individuals are currently living with Alzheimer’s disease and related dementias (ADRD) globally [1], with a significant increase projected over the next one (~75 million by 2030) and two decades (~130 million by 2050). ADRD is one of the world’s most expensive health conditions. Lifetime costs of care for individuals with ADRD are estimated at US$350,000 in 2018 dollars [2]. In the US, costs of ADRD are projected to grow from $290 billion in 2019 to more than $1 trillion in 2050 [2]. This number is doubled when estimating the global cost [3]. Given the magnitude of the problem in terms of individuals affected and the costs associated with ADRD, the World Health Organization recently asserted that prevention and treatment of ADRD is a public health priority [4]. 

Currently, no pharmacological disease modifying therapies (DMT) exist for the prevention or treatment of ADRD [5]. The past three decades have seen repeated failures in clinical trials of pharmacological therapies targeting beta amyloid, one of the pathologies present in individuals with Alzheimer’s disease. Currently, over 100 drug compounds are in some stage of clinical evaluation. Nevertheless, the failure rate to date has tempered the enthusiasm of current compounds in the clinical trial pipeline. While sobering, these failures have resulted in greater understanding and appreciation of the heterogeneity of ADRD, and the numerous genetic, biological, and behavioral factors associated with ADRD. At the same time, a growing body of research demonstrates the possibility for enhancing cognitive reserve and reducing risk of ADRD. These studies have consistently shown that ADRD are multifactorial in nature, with numerous genetic, environmental, and behavioral factors conferring protection or risk for ADRD. Given the large number of modifiable risk factors, including physical inactivity, poor diet, smoking, low education, midlife hypertension, midlife obesity, diabetes mellitus, depression, and education and occupational attainment [6], delay or prevention of ADRD through enhancing cognitive reserve and reducing modifiable risk factors offers a potential non-pharmacological DMT to reduce the growing number of individuals living with ADRD.

### From Single-Domain to Multi-Domain Interventions

**Figure 1:**
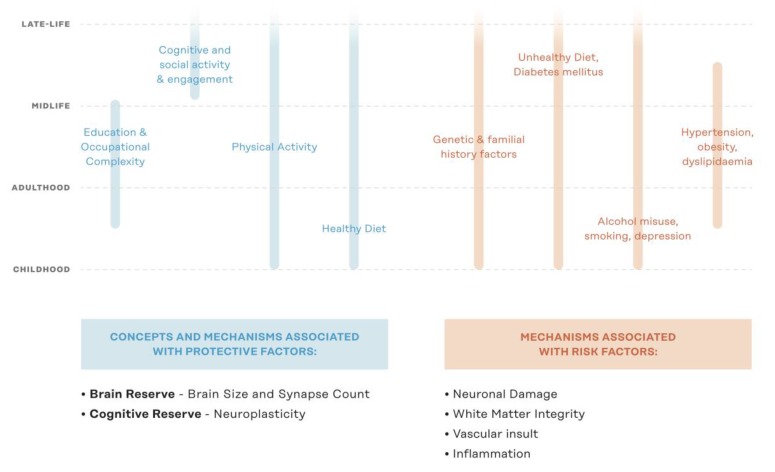
Lifespan protective and risk factors for ADRD. ADRD = Alzheimer’s disease & related dementias; Figure adapted from Kivipelto et al., 2018 [19].

Non-pharmacological trials aimed at ADRD risk factor reduction emerged in the early 2000s. These clinical trials focused on single domain interventions such as physical exercise [7], cardiovascular health [8], and cognitive training [9]. While some of these studies provided positive results, the heterogeneous nature of ADRD rendered many single-domain interventions ineffective when deployed in large randomized control trials [10,11,12,13]. The development of non-pharmacological interventions for reducing risk of delirium among older adults provides a useful analogy. Non-pharmacological interventions evolved from single-domain interventions—Originally deployed to treat older adults with delirium and later investigated for risk reduction—Into multidomain non-pharmacological risk reduction interventions for delirium [14]. This evolution came from the recognition that single-domain treatments were not efficacious, in combination with an understanding that multiple factors contribute to the onset of delirium and prevention is the most effective strategy for reducing the occurrence of delirium [15]. Given the heterogeneous nature of pathological cognitive decline and ADRD, several modifiable risk and protective factors exist at different points across the lifespan (Figure 1). Multidomain non-pharmacological interventions provide an opportunity to address the multiple risk factors simultaneously present among older adults at risk of ADRD. Over the past decade, the first large randomized control trials deploying multidomain non-pharmacological lifestyle interventions have been completed and have provided an initial evidence base for the efficacy and potential of these types of interventions to effectively enhance cognitive reserve and reduce risk of ADRD in specific older adult populations [16,17,18].

This study provides a comprehensive overview of completed and prospective non-pharmacological multidomain lifestyle interventions that aim to enhance cognitive reserve and reduce risk of ADRD. The review summarizes participant and intervention characteristics, study length and intervention frequency, primary and non-primary outcomes, adherence, and attrition. It also synthesizes studies completed to date and discusses how current and prospective studies are incorporating data reported from completed studies to further refine and optimize FTF and digital multidomain interventions.

## 2. Methods

A literature search utilizing PubMed, PsycInfo, ClinicalTrials, and NIH RePORTER was conducted from inception to August 1, 2019. Following previously utilized criteria, to be considered in the current review the study or protocol was required to meet a set of inclusion criteria associated with qualification as a multidomain lifestyle intervention to enhance cognition and reduce risk of ADRD [19]. First, the use of a control group was required to ensure scientific rigor. While many of the lifestyle behaviors that make up interventions in these studies have shown to be beneficial, the use of a control group is clinically important in discerning the true effects of the intervention. Second, interventional studies were required to contain 3 or more separate active lifestyle behavior components. This criterion was developed utilizing the FINGER protocol study design [17]. The FINGER protocol multidomain intervention included active behavior change across the domains of diet (healthy Nordic diet), physical activity (aerobic and strength training), and cognitive engagement (computerized cognitive training). In addition to these three active intervention components, intervention participants also received clinician feedback and motivation on the importance of managing vascular risk factors, as well as organic social engagement with other study participants. Given the difficulties in demonstrating significant and sustained cognitive outcomes in previous single-intervention studies [10,11,12,13,20,21,22], the FINGER protocol design provides a plausible heuristic for multidomain intervention development. Third, the intensity, frequency, and duration of intervention implementation in combination with maintenance and tracking was required to be at least 6 months. Studies exploring lifestyle change have noted that 6 months is generally the minimum length of time for participants to benefit from a positive lifestyle intervention due to the inherent difficulty of integrating these changes into daily practices and the nature of the behaviors being that are addressed [23]. This time frame also allows for the detection of possible cognitive benefits from the intervention, as changes to cognition are mostly seen over long periods of time [24]. Fourth, interventional studies were required to include measures of cognition as a primary or secondary outcome. Completed and prospective studies—Both face-to-face (FTF) and digital—Are also investigating the effects of multidomain behavioral interventions and monitoring on aspects of physical health and validated dementia risk measures [25,26]. While these studies are valuable in their own right, changes in cognitive performance between intervention and control participants strengthen the outcomes by which the efficacy of multidomain interventions can be evaluated. Furthermore, given that measurement of cognition is a requirement in the clinical determination of pathological cognitive decline (e.g., MCI or dementia), measures of cognition are needed to allow for the objective measurement of stability or change (either positive or negative).

## 3. Results

### 3.1. Completed Face-to-Face (FTF) Multidomain Interventions

#### 3.1.1. Prevention of Dementia by Intensive Vascular Care (preDIVA)

The preDIVA study (ISRCTN29711771) was a Dutch cluster-randomized control trial that evaluated the effect of a nurse-led multidomain intervention on cardiovascular risk factors and its effect on dementia incidence and disability in a sample of community-dwelling older adults aged 70–78 years [18,27]. Recruitment for this study was conducted at 26 healthcare sites in the Netherlands, with a total of 3526 participants. Over the course of 6 years, participants in the intervention group were assessed at their general practice site every 4 months, during which a nurse would assess smoking habits, diet, physical activity, weight, and blood pressure. Blood glucose and lipid concentrations were also assessed on a 2-year basis. Nurses provided individually tailored lifestyle advice, backed by motivational interview strategies, that incorporated Dutch general practitioner guidelines. Nurses also attended five information sessions that helped consolidate guidelines and standardize care practices across study sites. If needed, drug treatment for onset of cardiovascular disease (CVD) would be initiated or optimized for each participant. Primary outcomes were assessed using diagnostic criteria for dementia and Academic Medical Center Linear Disability score collected at follow-up periods. Information including cardiovascular disease onset, mortality, cognitive decline, and blood lipid concentration were collected for secondary outcome analysis. Of the 3454 (98.0%) of participants with full data and 3519 (99.8%) participants with survival data collected for the entire intervention period, dementia onset was seen in 121 (7%) of participants in the intervention group and 112 (7%) in the control group. In planned analysis, intervention participants did not vary significantly from controls in dementia onset (6% and 7%, respectively), dementia onset with baseline hypertension (5% and 7% respectively), mortality (16% and 16%, respectively), and cardiovascular disease events (19% and 17%, respectively). Participants with untreated hypertension and history without cardiovascular disease did show between group differences (4% and 5% for intervention against 7% and 7% for controls, respectively). Disability, cognition, and depressive symptoms did not differ between groups, and slight differences in systolic blood pressure, body mass index (BMI), total/low-density lipoprotein (LDL) cholesterol, and mortality were found to be non-significant between groups. While no overall effect was observed based on the intervention, this may be explained by the small difference between cardiovascular risk management strategies for the two participant groups due to a high standard of risk management in general practice. The older age of participants may have also contributed to the lack of significant effects between intervention and control groups [19].

#### 3.1.2. The Multidomain Alzheimer’s Prevention Trial (MAPT)

Utilizing a randomized four-arm placebo control design, the MAPT study investigated the efficacy of a dietary supplement and a multidomain intervention strategy (both in isolation and combination) versus placebo control for the prevention of cognitive decline [16,28]. Participants were instructed to take two pill capsules, containing either the omega-3 supplement or a placebo, throughout the duration of the study. For the multidomain intervention groups, participants completed 2-h sessions focusing on cognitive stimulation, physical activity, and diet on a twice per week basis for the first two months of the study. For the remainder of the three-year study, participants attended a monthly 1-h session reinforcing lesson, as well as a 2-h reinforcement session at 12 and 24 months. Adherence to interventions was assessed on a 6-month basis, using counts of capsules returned and sessions attended, as well as measures of omega-3 concentration in red blood cell membranes. Efficacy of the intervention was measured using a composite standardized score (*z*-score) of multiple cognitive tests (free/total recall of the Free and Cued Selective Reminding Test, ten mini mental state exam (MMSE) orientation items, Digit Symbol Substitution Tests, and Category Naming test). Individual components of this primary measure made up secondary measures, along with the Short Physical Performance Battery, Activities of Daily Living Prevention Instrument, Clinical Dementia Rating, frailty, and geriatric depression scale (GDS) scores. Of the 1268 (77%) participants that completed the study protocol, the multidomain-plus supplement group showed a slight increase in the primary composite score compared to a decrease in score from the placebo group, although these differences were found to not be significant. Three-year differences between baseline scores for multi-domain plus placebo and placebo group and baseline score for the supplement group and placebo group were also shown to be non-significant. Between group comparisons of secondary outcomes showed a slight difference for primary outcome components (only significant between multidomain + supplement vs. placebo). Nevertheless, the effects of the multidomain behavioral interventions (with or without omega 3 supplementation) on cognition—While not statistically significant—demonstrated trends toward early and sustained effects [29].

#### 3.1.3. The Finish Geriatric Intervention Study to Prevent Cognitive Impairment and Disability (FINGER)

The FINGER study was one of the earlier multidomain intervention studies carried out to investigate the prevention of cognitive decline and related disability in a population of at-risk older adult participants from across Finland [17]. This study recruited individuals based on a Cardiovascular Risk Factors, Aging, and Dementia (CAIDE) Dementia Risk score of at least 6 points [30,31,32], as well as mean/slightly below mean cognitive ability for their age. Cognitive ability was assessed using a number of neuropsychological test batteries completed before inclusion into the study [33]. Both control and intervention group participants received general health advice, physician examination at baseline and at the end of the active intervention (2 years), as well as visits with a study nurse to make general assessments (e.g., blood pressure and BMI) every 6 months after baseline. Advice on healthy diet and physical, cognitive, and social activities to manage cardiovascular and disability risk factors was given at baseline to all participants. The intervention group also received additional lifestyle interventions, including specific nutrition guidelines, exercise regimens, and cognitive training (both FTF and remote). Social interactions were facilitated through group meetings related to intervention components. Intervention participants also received vascular health monitoring with study nurses (at 3, 9, and 18 months) and physicians (3, 6, and 12 months). The outcome of interest was a composite score from an extended version of the neuropsychological test battery (NTB), used to assess general cognition [34]. Domain-specific cognition, vascular and lifestyle-related risk factors, depressive symptoms, dementia incidence, and disability were also tracked to assess the effect of the multidomain intervention on these outcomes. A total of 1105 (88%) participants completed assessments for the full duration, with 1190 (94%) of participants included in final analysis, demonstrating effective adherence techniques [35]. While both groups showed changes in mean NTB z-score, participants in the intervention group showed 25% greater improvement in NTB score than controls [36]. Intervention effects were also seen in improvements of executive functioning and processing speed, shown to be 83% and 150% higher than controls, and other secondary outcomes like BMI, diet habits, and physical activity. 472 (72%) of participants adhered to all intervention domains, and 52 (4%) participants reported adverse effects from intervention (mostly musculoskeletal pain). In addition to the primary composite cognitive outcome, a number of secondary outcomes have been have been reported, including health-related quality of life [37], reduced risk of chronic disease [38], maintenance of functional abilities [39], and intervention efficacy among different subpopulations characteristics [40], including ApoE e4 carrier status [41].

#### 3.1.4. The Systolic Blood Pressure Intervention Trial (SPRINT; SPRINT-MIND)

The SPRINT-MIND study was a sub-study of the SPRINT study to examine how intensive blood pressure interventions affect the incidence of MCI and dementia [42]. This RCT was conducted at 102 test sites in the United States and Puerto Rico with 9361 participants aged 50 years or older. Participants were either assigned into a group that aimed to attain systolic blood pressure (SBP) measures of <120 mm Hg (intensive) or <140 mm Hg (standard). Using an existing methodology outlined in the original SPRINT protocol [43,44], this trial delivered personalized treatment plans for participants including anti-hypertensive medication, weight-loss programs, dietary recommendations, and exercise plans. This was done to provide quality care in line with GP, as well as an effort to remove non-intervention changes to study outcomes. Participant SBP, as well as other measures, was measured monthly in the first three months of the trial and every three months for the rest of the trial. In addition, Intensive group participants would have a “Milepost” visit every 6 months to assess current treatments (e.g., if participant was at target < 120 mm Hg SBP) and change treatment plan if they did not meet expected measures. For the SPRINT-MIND extension participants took a number of cognitive assessment batteries and at baseline, 2 years, and 4 years into the trial and were classified into probable dementia (PD), mild cognitive impairment (MCI), or no impairment (NI) by a board of dementia specialists. The primary outcome for this trial was occurrence of PD in the sample, with secondary outcomes including occurrence of MCI and a composite of occurrence of MCI and PD. 3972 (92.6%) of intensive treatment and 3949 (92.3%) of standard treatment participants completed interventions through the original timeframe, with 2276 (61.1%) and 2191 (59.2%) participants from the groups completing assessments at extended timepoints, respectively. At extended follow-up, mean SBP was 129.2 mm Hg for intensive group and 135.6 mm Hg for standard group participants. PD incidence was seen in 149 and 176 cases for intensive group and standard group participants, respectively. MCI occurrence was seen less in intensive participants than standard participants (287 vs. 353 occurrences, respectively), but this difference was non-significant with further analyses. MCI/PD composite outcomes, on the other hand, showed significant differences between intensive and standard group participants (20.2 vs. 24.1 per 1000 person-years). While the interventions did not significantly reduce incidence of PD, this study provides a critical proof point in the delivery of a blood pressure intervention on cognitive status, as well as brain volume and white matter integrity [45,46], (Table 1).

### 3.2. On-Going and Prospective Face-to-Face (FTF) Multidomain Interventions

#### 3.2.1. Age Well.de

The Age Well.de study plans to further the research of multidomain interventions for cognitive impairment by offering a multi-center study with older-adult German participants. This ongoing study is investigating the feasibility of multi-domain interventions involving diet, physical activity, cognitive training, and vascular risk factors, and also includes recommendations for social lifestyle and medicine underuse/overuse [47]. Specific interventions will also be provided in cases of bereavement, grief, and depressive symptoms. Participants will be recruited from a group of community-dwelling general practitioner patients aged 60–77 years with a CAIDE Dementia Risk Score of ≥ 9. After recruitment from 4 general practices (*n* = 1152), participants will be separated into a group with advanced care (intervention) and a group with basic care and general health advice (control). The intervention program includes diet, exercise, cognitive training through tablet computers, and social engagement programs. It also offers participants assessments for depressive symptoms/risk factors, ways to assess and reduce vascular risk factors, and optimized medication plans if needed. Fully structured interviews will occur at baseline and 24-months for both groups, with the intervention group receiving an additional visit at 12 months to improve motivation and adherence to the program. The participants will also receive 5 phone calls throughout the course of the intervention to support adherence. This will look for differences in outcomes between the control and intervention group, using change in cognitive performance measured using a composite of *z*-scores from multiple neuropsychological batteries. The investigators will also track secondary endpoints such as mortality, depressive symptoms, and daily living, as well as readiness for behavior change in participants.

#### 3.2.2. Systematic Multi-Domain Alzheimer’s Risk Reduction Trial (SMARRT)

The SMARRT study plans to consolidate practices used commonly within multidomain intervention studies and compare outcome data to participants receiving generalized health education among older adults in the U.S. [48]. The SMARRT protocol will investigate whether a systematic multidomain intervention has a meaningful impact on cognition through changing personal risk factors for cognitive decline in intervention participants. Preliminary data will be gathered on an intervention that increases exercise, protective mental and social activities, and cardiovascular risk factor management, as well as reduction of depressive symptoms and use of contraindicated medication. This protocol also seeks to improve sleep behavior and introduce a neuroprotective diet. Participants will be recruited from 200 Kaiser Permanente patients aged 70–89 with slightly decreased cognition, as assessed through telephone-based cognitive screening, and at least two risk factors (e.g., poorly managed cardiovascular risk factors, high depressive symptoms, etc). Initial eligibility for the study will be identified using EHR data. Change in cognitive function will be measured using the modified Neuropsychological Test Battery that will be administered at 6-month intervals during the 2-year design, along with a number of surveys and incident scores to detect changes to the secondary outcomes mentioned above. After baseline assessment, intervention participants will also receive a FTF session in which personal risk factor areas will be addressed. SMARRT program participants will then form goals and be given tools to track progress, and subsequent meetings will be used to review intervention barriers and set new goals. Generally, intervention participants will have a phone or FTF contact session once a month, with FTF interventions happening at least twice a year. Control participants will receive packets in the mail with information on all risk factors targeted in the SMARRT intervention. 

#### 3.2.3. The Multimodal Preventive Trial for Alzheimer′s Disease (MIND-ADmini)

The MIND-ADmini study (NCT03249688) plans to use established multidomain intervention strategies to assess the efficacy of these interventions in a sample of 150 older adults aged 60–85 diagnosed with prodromal Alzheimer’s disease as defined by 1 standard deviation below age-based norms on 2 measures of cognitive function, including at least 1 measure of memory. The study is currently being conducted at sites in Sweden, Finland, Germany, and France using participants aged 60-85 years with preexisting cognitive impairment (excluding dementia). Adopting many practices used in the FINGER protocol, the MIND-ADmini will use three intervention arms. One will receive regular health advice (Control), one will receive FINGER-style interventions (Multidomain 1), and one will receive FINGER-style interventions with a multi-nutrient product to supplement diet (Multidomain 2). The primary outcome of this trial is the feasibility of the intervention, but adherence to intervention and lifestyle changes will be assessed as well. Cognition, independent living, and dementia scores will also be gathered to develop estimates for a larger future study (MIND-ADmaxi). This study will primarily focus on determining how successful adherence and intervention retention is. The MIND-ADmini will also build on past works with prodromal AD participants using the “medical food” supplement by seeing how its use may complement FINGER-style interventions. A 6-month extension to this study will also be considered once the original time frame has been completed.

#### 3.2.4. The Multiple Nonpharmacological Interventions Study (EmuNI)

The EMuNI study (NCT03382353) will break down popular components from a number of lifestyle intervention studies to look at specific effects of intervention strategies on cognitive performance endpoints and MRI markers for progression of Alzheimer’s Disease. Interventions on diet and lifestyle have shown to help with the onset of cognitive decline, and the EMuNI study plans to administer these interventions with increasing intensity. Control (no treatment, NT) participants will learn about cognitive domains through educational lessons and videos, partial treatment (PT) participants will consume a dietary supplement and receive nutritionist led lessons on a brain-healthy diet, and full treatment participants (FT) will receive the PT interventions along with supervised exercise and cognitive training sessions. This study is recruiting Italian adults aged 60–80 years with existing memory complaints and aims to determine if the combination of lifestyle interventions over the course of one year will lead to improvement in cognitive performance and positive magnetic resonance imaging (MRI) markers (e.g., hippocampal volume). Additionally, the investigators hypothesize that the combination of interventions will increase positive outcomes such that PT participants show more positive results than NT, and FT participants showing the most positive outcomes. 

#### 3.2.5. Taiwan Multidomain Intervention Efficacy Study—National Taiwan University Hospital

Cognitive decline often progresses to clinical levels before the proper steps to prevent or delay further decline can be taken. While many multicomponent studies investigate interventions in at-risk populations, this design plans to test the efficacy of these interventions in clinical populations. Using an equal number of participants with SCD and MCI, this study (NCT04023032) will collect 16 weeks of historical control data before the participants receive a multidomain cognitive intervention. The intervention includes cognitive training and cognitive rehabilitation, which aim to restore cognitive functions and providing strategies to support activities of daily life. Lifestyle and psychological interventions are also included, ranging from providing information on ways to balance risk factors (e.g., diet, exercise, cognitive training) and targeting common neuropsychiatric symptoms in SCD and MCI. These interventions will be carried out in weekly group sessions and 2 individual sessions over the course of 4-months, totaling 16 90-minute sessions. Participant status will be assessed using change in measures of memory capabilities and activities of daily living, as well as scores on individual cognitive batteries and anxiety/depression assessments.

#### 3.2.6. The Body, Brain, Life—General Practice Lifestyle Modification Program Study (BBL-GPLMP)

Another planned extension of the Brain, Body, Life studies, this trial will examine the effects of a lifestyle intervention plan on reducing dementia risk, specifically when a more thorough intervention is compared alongside a more standard approach [49]. This study will compare outcomes for a group in a standalone Lifestyle Modification Program (LMP) that receives 6 weeks of group education sessions, to a group with an integrated General Practice (GP) intervention. GP group participants will receive 12 weeks of tailored online education sessions and 1-h meetings with diet and exercise specialists to develop an intervention that best fits their needs. The study plans to recruit a total of 240 Australian adults with chronic health conditions (e.g., heart disease) or that are overweight/obese. The primary outcome measure will be Alzheimer’s Disease risk factor, calculated using the shortened version of the Australian National University – Alzheimer’s Disease Risk Index (ANU–ADRI) battery [50]. This trial will also look at a number of health-related outcomes, as well as depressive symptoms, diet/sleep quality, and cost-effectiveness. The GP intervention incorporates many methods from past programs in the Brain Body Life (BBL) initiative that demonstrated positive outcomes for participants, adapted for use with a wider age range. This trial hopes to demonstrate that this intervention designed for general use will lead to reduced dementia risk without the need for a stand-alone program or clinical research setting, (Table 2).

### 3.3. On-Going and Prospective World Wide Fingers Studies

The promising results reported on from the FINGER study has resulted in an initiative to explore the applicability of similar interventions across unique countries and cultures. Founded by Dr. Miia Kivipelto, the principal investigator of the FINGER trial, the World Wide FINGERS hopes to create an international network in which the key aspects of the FINGER trial can be replicated in countries around the world [51]. This global network of non-pharmacological intervention trials will allow researchers to share data and implement findings at an accelerated rate. World Wide FINGERS also allows for countries to explore cultural differences in necessary interventions while still contributing to a larger body of work [19]. Currently, this initiative supports multi-domain intervention studies in the U.S., China, Singapore, and Australia, with other countries joining the initiative and planning trials.

The U.S.-based outreach of the WW-FINGER initiative, the POINTER study (NCT03688126) will take the practices of the FINGER trial and test whether a similar intervention, tailored to U.S. culture, can help protect against cognitive decline in at-risk adults. This program will utilize a Structured Lifestyle Intervention which involves a program addressing diet, physical and cognitive exercise, and managing cardiovascular risks. Control participants will be given a self-guided intervention in which education, support, and tools to manage lifestyle practices are provided to participants. While many major disease cases will exclude participants from this study, the study is primarily seeking participants with poor diet, low levels of physical activity, and a direct family history of significant memory impairment. This is to purposefully include a sample that would uniquely benefit from lifestyle interventions. Participants will be recruited near testing sites in North Carolina and California. This study will explore the usefulness of this intervention strategy in a U.S. sample, as well as changes in global cognition and general health targeted by the intervention program.

The MIND-CHINA study aims to determine the effect of a multidomain lifestyle intervention adapted for Chinese culture on cognitive outcomes. This project, developed in collaboration with developers of the FINGER protocol, will utilize a similar multidomain intervention to the FINGER protocol in a sample of older, rural community-dwelling Chinese adults. Participants will be randomized into a cardiovascular risk factor management program (active control) or a cardiovascular risk factor program with additional multidomain lifestyle interventions. This intervention will be developed keeping cultural and lifestyle differences in mind, utilizing multi-disciplinary researchers from the Shandong Provincial Hospital to provide a proper intervention for rural Chinese adults.

The SINGER initiative is a trial that will test the efficacy and ease of implementation of a FINGER-like intervention for older adults in Singapore. As 20% of the population in Singapore is age 65 or older, this study will help elucidate if this type of intervention could benefit older Singaporean adults. Currently, a 6-month feasibility study is being conducted with a sample of 150 Singaporean older adults with MCI. This project is currently underway, and study investigators are working closely with investigators from the FINGER trial to develop a culturally sensitive intervention to help prevent future cognitive decline.

Additionally, two more country or region-specific deployments of FINGER are under way. The Australian-Multidomain Approach to Reduce Dementia Risk by Protecting Brain Health with Lifestyle intervention (AU-ARROW) is a 2-year intervention trial replicating the US POINTER study with an additional 6-month follow-up timepoint to assess sustainability of potential benefits. The GOIZ-ZAINU study is a pilot, controlled, randomized, one-year multimodal interventional study launched in June 2018. This study is adapting the FINGER protocol for the social and cultural context of the Basque population. Two hundred adults aged 60 or older will be enrolled with a CAIDE Dementia Risk Index score of 6 points or higher and below-expected performance on one brief cognitive screening task. Exploratory analyses will investigate reduction in risk scores and objective cognitive [52], (Table 3).

### 3.4. On-Going and Prospective Multidomain Interventions

#### 3.4.1. The Maintain Your Brain Study (MYB)

The Maintain Your Brain (MYB) study aims to reduce cognitive decline in a population of older Australian adults using a multimodal lifestyle intervention strategy administered exclusively through an online platform [53]. This study, while closely related to other studies linked to the WW-FINGERS initiative, offers a unique approach through a digital intervention. Rationale for this online approach stems from limitations of clinic attendance and scalability seen in other lifestyle interventions. The MYB platform includes four modules that engage risk factors for dementia, including physical activity, diet, cognition, depression, and anxiety, and a number of health-related issues (e.g., smoking, obesity, alcohol consumption). A unique aspect to the approach taken in this study is the customization of intervention, as required program modules only reflect the risk factors for a particular participant. Participants in the intervention group will receive information about these topics—Dubbed “Physical Activity”, “Nutrition”, “Peace of Mind”, and “Brain Training”—As well as personalized coaching to instruct participants and help overcome barriers in progress [54]. Participants will be given 2–4 modules to complete in the first year (based on their individual assessments), followed by quarterly booster sessions and annual follow-up sessions. This approach will shed light on the effects of targeting specific risk factors rather than utilizing a “one-size fits all” program. In comparison, “information-only” participants will only receive the information without individual coaching to see how the personalized approach changes outcomes. Participants will be recruited from the 45 and Up Study, a cohort of 267,153 individuals aged 53 + and older from New South Wales, Australia. The investigators aim to recruit 2143 individuals for each study arm (with an assumed 20% dropout rate). The primary outcome is change in cognition following three years, measured using a composite of multiple cognitive test *z*-scores included in the “MYB Battery”. Secondary outcomes span a number of domains, including dementia incidence/cognitive impairment, intervention impact, and impact on module-targeted risk factors.

#### 3.4.2. The Digital Cognitive Multidomain Alzheimer’s Risk Velocity Study (DC-MARVEL)

While existing FTF interventions have demonstrated meaningful participant engagement, these studies remain geographically constrained and require a significant amount of labor [55]. A number of factors can prevent individuals from having access to opportunities for such interventions. One solution is to use a fully digital intervention strategy that can be widely implemented with ease. The DC-MARVEL trial aims to address the scalability of non-pharmacological lifestyle interventions through a fully digital app-based multidomain intervention. The Digital Cognitive Multi-domain Alzheimer’s Risk Velocity (DC-MARVEL) trial (1R44AG063672-01) will provide lifestyle interventions using a digital therapeutics app-based platform developed using the FINGER protocol as a framework. The interventions offered through this app-based platform have been examined in a single-arm pilot study with older adults experiencing varying degrees of subjective cognitive decline. Recruitment, screening, and assessments in the pilot study were done entirely using remote and online methods [56,57]. At the end of the 52-week intervention, participants showed increased scores on measures of cognition and decreased measures of depressive symptoms. Many participants also expressed that they were engaged and satisfied by interventions offered featured in the program [58]. For the DC-MARVEL trial, 200 at-risk adults will be randomly assigned to a digital therapeutic Memory Health Program (MHP) that will be compared to a digital health education program (control). Outcomes will be measured in 2-year difference from baseline on ANU-ADRI and composite cognitive performance (total score of the Repeatable Battery for the Assessment of Neuropsychological Status), as well as change in clinical biomarkers, behavioral symptoms, and quality of life measures. This trial aims to further the study of digital remote solutions designed to help address a large at-risk population that would otherwise not have access to clinical interventions.

#### 3.4.3. The Body, Brain, Life for Cognitive Decline Study (BBL-CD)

Building on a series of works, the BBL-CD trial is the most recent iteration of a multidomain dementia risk factor intervention program that has been adapted for participants with cognitive impairment [26,59,60]. This study modified approaches taken in other BBL studies by reducing the number of information modules received by participants (4 modules in 8 weeks), with a 1-week gap to incorporate new information into their lifestyles. Participants will then be reassessed immediately following the intervention (week 9), then again at 3 months (week 20) and 6 months (week 32). Learning modules include an introduction to dementia literacy (week 1), diet (week 2), cognitive engagement (week 4), and physical activity (week 6). While this information will be given to both the intervention group and control group participants, the intervention group will also receive “practical components” reinforcing the incorporation of this knowledge into daily living. The intervention group also receives online cognitive batteries and face-to-face meetings with specialists (dietitians and exercise physiologists) to make personalized intervention plans. Follow-up meetings with these specialists will also happen twice after initial reassessment (10 and 21 weeks). All participants will receive newsletters with information relevant to the completed modules, as well as a summary of points at the end of the module course. To monitor the prevention of cognitive decline, the BBL-CD will use ADAS-Cog-Plus to track standard cognition, executive function and ADLs, and ANU-ADRI to assess AD risk factors and protective lifestyle factors. This study will also track motivation, health-related quality of life, BMI, and adherence measures, as well as measures directly related to the modules. While the intervention period in this study is short, it incorporates many strategies that have been shown to help with cognitive decline in past studies with a sample that is currently experiencing cognitive decline.

#### 3.4.4. Healthy Ageing Through Internet Counselling in the Elderly (HATICE)

The HATICE trial was developed to address a number of modifiable lifestyle-related and vascular risk factors found in cases of dementia and cardiovascular disease (CVD) in older adults [61,62]. The HATICE trial was conducted in The Netherlands, Finland, and France to assess the efficacy of implementing these interventions across Europe. This study focused on reducing cardiovascular risk factors by introducing exercise and diet recommendations, as well as smoking prevention/rehabilitation, that were supported remotely by a coach throughout the study in the intervention arm. Changes were measured using a composite score based on body-mass index (BMI), systolic blood pressure (SBP), and low-density lipoproteins (LDL) measures. The intervention was originally carried out with 41 participants in a trial run, carried out in part to investigate efficacy of the intervention methods across European populations. While slight differences existed between information and diagnostic criteria due to country-specific guidelines (e.g., higher recommended alcohol intake in French guidelines vs. Dutch and Finnish guidelines), interventions were generally uniform. One major difference between guidelines was found in assessing overall cardiovascular risk, but this difference did not affect the intervention overall due to country-specific adaptations to the intervention to maximize effectiveness. This study demonstrated that with some consideration to how cardiovascular risk is assessed in different countries, HATICE-style multidomain intervention could be implemented internationally to help prevent risk factors associated with CVD and dementia. Data has been collected for 2725 participants but the study is still underway, (Table 4).

## 4. Conclusions

Alzheimer’s disease and related disorders (ADRD) pose population health risks to virtually every society around the world. The health, societal, and economic burdens associated with ADRD have transformed ADRD into a global health priority [1,4,63,64]. The lack of pharmacological-based disease-modifying therapies (DMT) to date [65], combined with the low likelihood of effective pharmacological DMTs in the near future [5], has brought increased focus to non-pharmacological reserve and risk reduction (RRR) interventions [66,67]. Over the past three decades, mounting evidence has linked multiple lifestyle factors with increased risk of pathological cognitive decline, including ADRD [68,69,70,71,72,73,74]. This evidence has been complemented by epidemiological data, indicating that declines in dementia incidence are likely due to better management of lifestyle behaviors [75,76,77].

The existing evidence base from completed multidomain RCTs points to the clinical utility of non-pharmacological, lifestyle-based interventions for enhancing cognitive reserve and reducing risk of ADRD. At the same time, these studies point to the necessity of multidomain interventions to successfully address the multiple risk factors associated with ADRD in aging populations. One of the enduring challenges in the development of pharmacological DMTs for ADRD has been intervening at the point in disease progression that is clinically efficacious [78]. To date, trials targeting individuals with detectable cognitive or cognitive and functional impairment have failed [65]. Given the underlying pathological processes at work that can precede readily observable clinical symptoms, current pharmacological trials are now targeting pre-clinical populations [79,80]. Owing to the multifactorial etiology of ADRD that occurs dynamically across different life stages, multidomain non-pharmacological lifestyle interventions also need to utilize a life-course approach. Cardiovascular health factors provide an example of the dynamism involved. Vascular risk in ADRD, often focused on SBP and blood cholesterol, are targets for middle-aged individuals with risk for ADRD. Among older adults, less is known with respect to optimization of these factors [81]. Similarly, social isolation has been associated with Alzheimer’s disease pathology and MCI [82,83]. Interventions that target social engagement, however, may not be optimal until past middle age. 

The unique use cases for non-pharmacological multidomain interventions also warrant consideration. Within clinical research contexts, the use of clinical diagnostic criteria such as the recently adopted NIA-AA criteria for asymptomatic or pre-clinical Alzheimer’s disease [80] provide for highly characterized study populations. At the same time, Alzheimer’s disease pathology rarely occurs in isolation from other forms of pathological burden (e.g., vascular pathology) [84] with interactions between pathologies posited in the initiation and accumulation of pathological burden [85]. Given this heterogeneity, SCD and subtle, measurable changes in cognitive performance reported by patients remains a critically important signal for initiation of reserve building and risk reduction interventions.

As multidomain intervention studies are now being implemented across the globe, effective localization of these interventions will grow in importance. To date, the evidence base for these studies has occurred in high-income countries, with substantial infrastructure and clinical workforce for the deployment of these programs. Yes, it is clear that middle- and lower-income countries will be adversely affected by ADRD in the coming decades [86,87,88]. Tailoring of multidomain interventions across geographic, cultural, and economic factors will be increasingly important to maintain efficacy and adoption across diverse contexts.

A unique opportunity exists for digitally-mediated multidomain intervention programs to reach populations in low- and middle-income countries in ways that physical infrastructure and clinical work forces cannot [89,90]. Digitally deployed interventions, in combination with support through health coaching, remove geographical barriers and offer opportunities for individuals to participate with others even in remote or isolated contexts. Moreover, digital deployments of these programs are uniquely positioned to augment FTF programs as a means of reinforcing intervention components, maintain engagement, and support effective adherence for optimal dosing of unique lifestyle behaviors. Given the rise of digital therapeutics to address chronic disease, digital multidomain intervention programs to enhance cognitive reserve and reduce risk of ADRD are well-positioned to effect lifestyle change in at-risk individuals with both short- and longer-term health benefits [38,39,40].

A common challenge to both FTF and digital multidomain intervention programs is adherence. The FINGER study reported high engagement (7% dropout at 12-months) with variable adherence across individual intervention components (e.g., >90% for cardiovascular monitoring, <50% for cognitive training). Similarly, adherence in the MAPT study was variable (e.g., >75% for Omega-3/placebo tablets, ~60% adherence to multidomain sessions and cardiovascular consultations) [35]. Adherence to digitally delivered programs is an important area of current research [91,92], with evidence supporting the role of content and intervention tailoring to specific populations and individuals to improve adherence [93,94]. Additionally, the use of health coaching within digitally delivered programs can further influence motivation, engagement, and adherence [95]. Multidomain interventions targeting the enhancement of cognitive reserve and reduction of risk factors associated with ADRD enjoy a unique position due to the numerous non-dementia specific outcomes that these programs can effectively address in older adult populations. A “top-down” approach to lifestyle interventions, beginning with cognitive health, has demonstrated improvements in cognition [36], health-related quality of life [37], self-perceived physical function and general health [39], and chronic disease risk reduction [38].

The public health relevance of these non-pharmacological approaches is an important consideration. A shift in mindset from pharmacological cure to lifestyle care is needed in order to embrace the significant potential benefits of enhancing cognitive reserve and reducing risk of ADRD. Even small reductions in ADRD incidence will have outsized public health impacts. Given that as much as many as half of all cases of ADRD may be attributable to modifiable risk factors [96], non-pharmacological multidomain interventions provide a viable opportunity to effect population level health. Microsimulation models such as the Future Elderly Model have shown total societal savings of more than $100 billion from delaying disease onset by just one year in the US [97]. Similarly, epidemiological studies have reported that a large portion of the years lived with ADRD are amenable to population level risk reduction interventions, with even minor risk reduction yielding significant public health benefits [76,98] and may be cost-effective [99]. 

In the near future, the measurement and management of cognitive health from mid-life onwards will provide for proactive intervention to enhance cognitive reserve, reduce risk factors associated with pathological cognitive decline including ADRD, and optimize cognitive aging at the societal and population levels. For this to be achieved, a combination of FTF, brick and mortar multidomain lifestyle interventions, and digitally delivered multidomain interventions are needed. Digital interventions and digital therapeutics such as those reviewed here are critical adjuncts to FTF interventions that support engagement and adherence, while simultaneously serving as an effective stand-along delivery mechanism for evidence-based enhancement of cognitive reserve and risk reduction. In a similar way to how cardiovascular disease and diabetes mellitus are currently addressed at a population health level—With non-pharmacological lifestyle interventions as the first line intervention for both prevention and treatment—Multidomain lifestyle interventions are poised to be the first line intervention for prevention and treatment. Additionally, as effective repurposed or novel pharmacological DMTs emerge, drug-based interventions can be used in combination with lifestyle interventions to complement or increase efficacy utilizing a precision medicine approach. A dual therapy approach, including both pharmacological and non-pharmacological interventions, may represent the most optimistic future for the prevention and treatment of ADRD. However, while this optimistic future is not a reality, the reality of FTF and digital multidomain lifestyle interventions to enhance cognitive reserve and reduce risk of pathological cognitive decline provides much needed optimism and offers the potential to slow the growing number of individuals living with ADRD.

## Figures and Tables

**Table 1:** Completed Face-to-Face multidomain interventions to enhance cognitive reserve and reduce risk of Alzheimer’s disease and related dementias (ADRD).

Study Title	Study Sample	Intervention Components	Study Length & Intervention Frequency	Primary Outcomes	Other Outcomes	Adherence/Attrition	Limitations
FINGER	*n* = 1260 Finnish adults age 60–77 CAIDE score > 6 Average or slightly declined cognition	Diet Exercise Cognitive training Social activities Metabolic/vascular risk management	Daily diet plan Strength training 1–3x/week, aerobic 2–5x/week Cognitive training 3x/week (10 group sessions) Risk management checkup at 3 time points 2 year study, follow up at 7 years	Mean NTB score difference was shown to be 0.022 points (standardized) higher between groups (intervention vs. controls)	Significant intervention effect on executive function and processing speed Risk of cognitive decline increased for controls vs. intervention Significant intervention effects on BMI, exercise, and diet.	1190 (94%) participants used for final analysis 416 (72%) of intervention subjects completed all domain interventions regularly 86 (14%) intervention and 66 (11%) control participants dropped out; mostly health-related.	Participants may have had existing dementia-related changes to the brain. Providing necessary health knowledge to controls may masked the true intervention effect.
MAPT	*n* = 1680 French adults age ≥ 70 Spontaneous memory complaint Limitation in IADL Slow Gait	Diet Exercise Cognitive Training Management of cardiovascular risk factors Omega 3 dietary supplement (in 2 arms)	3 year study 12 two-hour sessions on cognitive training, physical activity, and nutrition 2 sessions per week in first month and 1 session per week in second month Preventive consultation at baseline, 12 months, and 24 months	Participants in multi domain + supplement intervention showed increase in cognitive score compared to placebo, but not significant.	Less decline in MMSE items used in composite for multi domain + supplement versus placebo.	1268 (77%) participants completed study	Did not look at individual contributions of components Low intensity of intervention
preDIVA	*n* = 3526 Dutch adults aged 70–78 years	Exercise Management of cardiovascular risk factors Smoking Cessation Health Coaching Chronic Disease Management	6 years 18 visits total (1x every 4 months) Individually tailored lifestyle advice and medication adjustment	No difference in incidence of dementia between groups at 6 years	No difference in disability, cognitive, or depressive symptoms. Non-significant differences between groups in systolic blood pressure, BMI, total/LDL cholesterol, and mortality.	3519 (99.8%) participants completed study	Small difference in risk between groups may be due to high standards of usual care.
SPRINT-MIND	*n* = 9361 American and Puerto Rican adults aged 50 + years Baseline systolic blood pressure between 130 and 180 mm Hg	Antihypertensive Medication Diet Exercise Weight loss	4 years (8 years for final follow-up) Biometrics collected every 3 months Cognitive batteries administered at baseline, 2, and 4 years “Milepost” assessment for intervention every 6 months	No difference in probable dementia occurrence between groups.	Difference in SBP measure for intensive vs. standard group Occurrence of MCI was lower in Intensive group, but not significant. Significant between-group difference in probable dementia/MCI composite (favoring Intensive).	3972 (92.6%) of intensive treatment and 3949 (92.3%) standard completed cognitive assessment at follow up Completion rates above 90% for both groups at 2 & 4 years	Intervention terminated early for cardiovascular benefits Loss of participants to follow-up may have lead to underestimated conversion to PD/MCI

The gray background is just to the table to be clearer. ADRD = Alzheimer’s disease and Related Dementias; BMI = body mass index; FINGER = Finnish Geriatric Intervention Study to Prevent Cognitive Impairment and Disability; IADL = Instrumental Activities of Daily Living; MAPT = Multidomain Alzheimer Preventive Trial; MCI = Mild Cognitive Impairment; MMSE = Mini Mental State Exam; NTB = Neuropsychological Test Battery; preDIVA = Prevention of Dementia by Intensive Vascular care; SPRINT-MIND = Sub-study of Systolic Blood Pressure Intervention Trial.

**Table 2:** On-going or prospective Face-to-Face multidomain interventions to enhance cognitive reserve and reduce risk of ADRD.

Study Title	Sample/Sampling Method	Interventions	Study Length & Intervention Frequency	Main Outcomes	Issues Addressed
Age well.de	*n* = 1152 German adults aged 60–77	Diet Exercise Vascular risk factor management Cognitive training Medication management Social lifestyle Depressive/Grief symptom management	2 years Structured interview at 12 and 24 months. Motivational meeting at 12 months for intervention group.	Improvement in composite Cognitive Score for intervention Decreasing mortality and depressive symptoms Keeping track of IADL and readiness for change through intervention	Lack of studies done with German populations Health complications over mismanaged medication Social support
SMARRT	Kaiser Permanente patients aged 70–89 Decreased cognition Minimum of 2 risk factors HER screening	Diet Exercise Mental exercises Social experiences Vascular risk factor management Medication management	2 years Cognitive assessments every 6 months Risk Assessment and Counseling following cognitive assessments	Change in NTB scores over time Improvement in all areas of intervention components Improve sleep quality and positive behavior	Looks at efficacy multi domain interventions in U.S. Possible negative medication interactions Developing goal-setting behavior post-study
EMuNI	Italian adults aged 60–80 Existing memory complaints	Cognitive health literacy Diet Nutritional supplement (Tramiprosate) Exercise Cognitive training	1 year Biweekly nutrition lessons Daily supplement Weekly exercise Biweekly cognitive training	Improvement in cognitive batteries Increase of positive MRI markers Increasing positive outcomes in more intense intervention groups	Explores how different intensity level interventions affect positive outcomes Includes participants with subjective cognitive decline
MIND-ADmini	*n* = 150 (estimated) Adults aged 60–85 Prodromal AD Score of ≥3 on Lifestyle Index MMSE ≥ 24	Diet Exercise Cognitive training Vascular risk factor management Nutritional Supplement (Fortasyn Connect)	6 months (with a possible 6-month extension)	Feasibility of/adherence to intervention Encourage lasting lifestyle change Positive cognitive and health related outcomes	Participants experiencing cognitive impairment Exploring use of dietary supplement paired with multi-domain intervention
Taiwan Multidomain Intervention Efficacy Study	Participants with MCI (*n* = 35) and SCD (*n* = 35)	Diet Exercise Cognitive training Smoking cessation Neuropsychiatric symptoms	1 year 16-week control data gathering Weekly intervention meetings for 4 months 2 individual sessions	Increasing cognitive battery scores Supporting ADL and targeting neuropsychiatric symptoms	Taiwanese Sample Participants experiencing cognitive decline
Brain, Body, Life: General Practice, Lifestyle Modification Program (BBL-GPLMP)	*n* = 240 (estimated) GP referred CHC or overweight	Exercise Diet Online psychoeducation modules	GP: 12 FTF sessions over 6 weeks LMP: 8 online sessions; 1 session each with dietician & EP	Validated AD risk factor survey Cognition, PA, depressive symptoms, diet, sleep quality	Intervention delivered within clinical workflow Australian sample

The gray background is just to the table to be clearer ADRD = Alzheimer’s disease and related dementias; EHR = Electronic Health Record; EP = Exercise physiologist; EMuNI = Efficacy of Multiple Nonpharmacological Interventions; IADL = Instrumental Activities of Daily Living; MCI = Mild Cognitive Impairment; MIND-ADmini = Multimodal Preventive Trial for Alzheimer’s Disease (Mini); MMSE = Mini Mental State Exam; NTB = Neuropsychological Test Battery; PA = Physical activity; SCD = Subjective Cognitive Decline; SMARRT = Systematic Multi-Domain Alzheimer’s Risk Reduction Trial.

**Table 3:** On-going and prospective World-Wide Fingers multidomain interventions to enhance cognitive reserve and reduce risk of ADRD

Authors/Date	Sample/Sampling Method	Interventions	Study Length & Intervention Frequency	Main Outcomes	Differentiating Factors from FINGER
POINTER	U.S. adults age 60–79 years *n* = 2000 (estimated) High risk from lifestyle factors (e.g., poor diet) First-degree family history of memory impairment	Diet Exercise Vascular risk factor management Social stimulation Cognitive training	2 years	Efficacy of multi domain intervention, culturally suited to Americans Protection from cognitive decline for high-risk individuals	U.S. sample High-risk individuals used to potentially show greater benefits of intervention
SINGER	*n* = 150	Diet Exercise Vascular risk factor management Social stimulation Cognitive training	6 months	Increased protection against cognitive decline Usefulness/ease of implementation for Singaporean adults	Singaporean Sample
MIND-CHINA	Rural Chinese adults aged 60–79 years	Diet Exercise Intellectual training Social activities Vascular risk management Lifestyle guidelines		Compare vascular risk factor treatment plans	Chinese sample

The gray background is just to the table to be clearer. POINTER = Study to Protect Brain Health Through Lifestyle Intervention to Reduce Risk; MIND-CHINA = Multimodal Intervention to delay Dementia and disability in rural China; MYB = Maintain Your Brain; SINGER = Singapore Intervention Study to Prevent Cognitive Impairment and Disability.

**Table 4:** On-going or prospective digital multidomain interventions to enhance cognitive reserve and reduce risk of ADRD.

Title	Sample/Sampling Method	Interventions	Availability	Study Length	Primary Outcomes	Issues Addressed
MYB	*n* = 2143 (planned) Australian adults aged 53 + Recruitment from longitudinal health study (45 and Up)	Exercise Diet Cognition Depressive/Anxiety symptoms Lifestyle risk factors (e.g., smoking/heavy drinking)	3 years 2–4 modules assigned in 1 year (risk factor dependent) Motivational session every 3 months Annual follow-up	Improvement/lack of decline in composite cognitive score Decreased incidence of dementia Impact on module-focused risk factors Assessing efficacy of an online approach	Fully remote intervention Personally tailored interventions	Web-based intervention Fully digital intervention Personalized risk-factor intervention
DC-MARVEL	*n* = 200 (planned) Aged 45–64 years At risk for dementia	Diet Exercise Cognitive training Sleep Stress Social engagement Health coaching	Online Not publicly available	2 years	Lifestyle risk and protective factor score Cognitive assessment score Clinical biomarkers	Cross-platform, app-based intervention Fully digital intervention Personalized intervention plans
BBL-CD	Australian adults aged 65 + years SCD or previously diagnosed MCI	Diet Exercise Cognitive activity	Online Not publicly available	6 Months 1 module/ 2 week (one week in between) Assessed at 9 weeks, 3 and 6 months	Cognition, Executive Function and IADLs (ADAS-Cog-Plus) AD risk/protective lifestyle factors Motivation, health-related quality of life, adherence	Personalized intervention plans Participants experiencing cognitive impairment
HATICE	*n* = 2725 Finnish, Dutch, French adults age 65 + Two or more cardiovascular risk factors History of diabetes or cardiovascular disease	Diet Exercise Cardiovascular risk factor management	Online, not publicly available	18 months FTF interview and biometrics at baseline and 18 months. Online questionnaires at baseline, 3, 12 and 18 months. Phone call for medication use at 12 months	Increase in composite z-scores of biometrics from baseline Intervention unaffected by cultural differences (when adjusted to that culture)	Culture-specific guidelines on CVRF/weight can affect implementation Coaches serve mostly as motivational support for change

The gray background is just to the table to be clearer. ADRD = Alzheimer’s disease and related dementias; BBL-CD = Body, Brain, Life for Cognitive Decline; DC-MARVEL = Digital Cognitive Multi-domain Alzheimer’s Risk Velocity study; FTF = Face-to-face; HATICE = Healthy Aging Through Internet Counselling in the Elderly; IADL = Instrumental Activity of Daily Living; MCI = Mild Cognitive Impairment; SCD = Subjective Cognitive Decline.
